# Therapeutic Effects of Neuro-Cells on Amyloid Pathology, BDNF Levels, and Insulin Signalling in APPswe/PSd1E9 Mice

**DOI:** 10.3390/cells14161293

**Published:** 2025-08-20

**Authors:** Johannes P. J. M. de Munter, Andrey Tsoy, Kseniia Sitdikova, Erik Ch. Wolters, Kirill Chaprov, Konstantin B. Yenkoyan, Hamlet Torosyan, Sholpan Askarova, Daniel C. Anthony, Tatyana Strekalova

**Affiliations:** 1 Department of Psychiatry and Neuropsychology, Maastricht University, 6229 ER Maastricht, The Netherlands; h.demunter@neuroplast.com; 2 Neuroplast B.V., 6222 NK Maastricht, The Netherlands; ech.wolters@gmail.com (E.C.W.); chapkir@gmail.com (K.C.); 3 Center for Life Sciences, National Laboratory Astana, Nazarbayev University, Astana 010000, Kazakhstan; andrey.tsoy@nu.edu.kz (A.T.); sitdikova.kk@gmail.com (K.S.); shaskarova@nu.edu.kz (S.A.); 4 Department of Neurology, University of Zurich, 8091 Zurich, Switzerland; 5 Neuroscience Laboratory, COBRAIN Center, Yerevan State Medical University, Yerevan 0025, Armenia; konstantin.yenkoyan@meduni.am (K.B.Y.); hamlettorosyan1@gmail.com (H.T.); 6 Department of Pharmacology, University of Oxford, Oxford OX1 3QT, UK; daniel.anthony@pharm.ox.ac.uk

**Keywords:** stem cell therapy, mesenchymal stem cells (MSCs), haematopoietic stem cells (HSCs), Alzheimer’s disease, APP/PS1 mice, insulin receptor, neurotrophins, amyloid plaques, emotionality, cognition

## Abstract

Stem cell therapies, including mesenchymal (MSCs) and haematopoietic stem cells (HSCs), have shown promise in neurodegenerative diseases. Here, we investigated the therapeutic effects of a defined combination of unmanipulated MSCs and CD34^+^ HSCs, termed Neuro-Cells (NC), in a murine model of Alzheimer’s disease (AD), the APPswe/PS1dE9 mouse. At 12 months of age, mice received intracisternal injections of NC (1.39 × 10^6^ MSCs + 5 × 10^5^ HSCs) or vehicle. After 45 days, behavioural testing, immunohistochemical analyses of amyloid plaque density (APD), and cortical gene expression profiling were conducted. NC-treated APP/PS1 mice exhibited preserved object recognition memory and reduced anxiety-like behaviours, contrasting with deficits observed in untreated transgenic controls. Histologically, NC treatment significantly reduced the density of small amyloid plaques (<50 μm^2^) in the hippocampus and thalamus, and total plaque burden in the thalamus. Gene expression analysis revealed that NC treatment normalised or reversed disease-associated changes in insulin receptor (IR) signalling and neurotrophic pathways. Specifically, NC increased expression of *Bdnf*, *Irs2*, and *Pgc-1α*, while attenuating aberrant upregulation of *Insr*, *Igf1r*, and markers of ageing and AD-related pathology (*Sirt1*, *Gdf15*, *Arc*, *Egr1*, *Cldn5*). These findings indicate that NC therapy mitigates behavioural and molecular hallmarks of AD, potentially via restoration of BDNF and insulin receptor-mediated signalling.

## 1. Introduction

Alzheimer’s disease (AD), the most common form of dementia, is a progressive neurodegenerative disorder characterised by gradual loss of neurons, resulting in cognitive decline and behavioural symptoms, in which amyloid accumulation is generally considered a pivotal mechanism [1,2]. Currently, there are only a limited number of disease-modifying therapies for AD, such as the recently approved lecanemab [3] and adducanumab [4], both targeting beta-amyloid levels. However, these treatments offer only modest neurological improvements and are limited by side effects and high costs [5]. Growing evidence supports the therapeutic potential of stem cells in AD. Various stem cell types, such as those derived from bone marrow, embryonic tissues, umbilical cord blood, or induced pluripotent stem cells, have demonstrated beneficial effects in preclinical and some clinical settings [6,7].

Among them, mesenchymal stem cells (MSCs) and haematopoietic stem cells (HSCs) have shown particularly promising neuroprotective effects through mechanisms that include modulation of Aβ metabolism, attenuation of neuroinflammation, enhancement of neurogenesis, and reduction in oxidative stress [8]. For example, transplantation of wild-type HSCs in AD mouse models has shown promising results, including the prevention of memory loss, reduction in β-amyloid plaques, and decreased neuroinflammation by suppressing microglial activation [9]. HSCs were suggested to realise their beneficial effects via a differentiation to endothelial and thereby contributing to a repair of vascular damage and an increase in brain blood barrier (BBB) permeability accompanying AD [10,11]. A large body of evidence has demonstrated the high therapeutic potential of MSC transplantation in AD. Preclinical studies using transgenic AD mouse models and in vitro systems have demonstrated that MSCs derived from bone marrow and other tissues enhance amyloid-β (Aβ) clearance and attenuate tau hyperphosphorylation [12].

The administration of MSCs has been shown to decrease the expression of tumor necrosis factor (TNF), to elevate levels of interleukin-10 (IL-10) and vascular endothelial growth factor (VEGF) [12], and to increase the expression of oxidative stress defense enzymes catalase and superoxide dismutase [13]. These cells are also shown to enhance neurogenesis by upregulating markers of neural proliferation and differentiation (doublecortin, Ki-67, and nestin) and promote Wnt signalling and the expression of neurotrophic factors, such as brain-derived neurotrophic factor (BDNF) and sirtuin 1, facilitating neuronal survival and synaptic integration [14]. Intravenously injected bone marrow-derived MSCs reduced amyloid formation and improved memory in animal studies [15,16]. Notably, bone marrow-derived stem cells secrete anti-inflammatory cytokines and trophic factors, including IL-10, BDNF, VEGF, insulin-like growth factor-1 (IGF-1), hepatocyte growth factor (HGF), and nerve growth factor (NGF), with secretion profiles that are modulated by the local inflammatory milieu [17,18].

These findings link the effects of stem cell therapy to inversely interrelated inflammation and neurotrophin-mediated signalling in AD conditions and raise a question about the possible implications of the latter in the mechanisms of action of stem cells in neurodegeneration. Several studies have suggested a role of regulatory processes mediated by the insulin receptor family in the beneficial effects of stem cell therapy on AD pathology [19,20]. Members of this family include insulin receptor (IR), BDNF receptor TrkB, and IGF-1 receptor (IGF-1R), all of which show high structural homology between their catalytic sites and activation loops, according to the UniProt/KB Swiss-Prot data bank [21]. These receptors and their ligands have been shown to be involved in multiple mechanisms of synaptic plasticity, cell survival, and regeneration [22,23].

Insulin resistance within the central nervous system and dysregulation of IR-related pathways, including IR substrate-1 (IRS-1), IRS-2, IGF-1, and BDNF, are well-established features of AD pathology [24,25,26]. Disruption of insulin signalling contributes to oxidative stress, mitochondrial dysfunction, and neuroinflammation [25,26]. Insulin is synthesised locally by neurons and astrocytes [27,28] and released in an activity-dependent manner [29], a process linked to Wnt/β-catenin pathway activation [30]. Neuronal insulin production has been demonstrated in GABAergic neurons of the cortex, hippocampus, and hypothalamus, and is particularly abundant in neural progenitor cells of the olfactory bulb and hippocampus [27,30,31]. These findings suggest that disruption of IR signalling may represent a key therapeutic target in AD and raise the question of whether stem cell therapies act in part by modulating this pathway. However, this has not been systematically addressed in experimental models.

“Neuro-Cells” (NC) is a unique stem cell preparation as it comprises non-manipulated stem cells that decrease the potential risks of its use, particularly in the elderly, and includes a combination of two cell types, MSCs and HSCs (bearing CD105^+^, CD90^+^, CD271^+^, or CD73^+^) and HSC (CD34^+^) isolated from the bone marrow. While the majority of clinical and preclinical observations use a single type of stem cell as a therapy for AD-related neurodegeneration, recent evidence suggests the advantages of combined stem cell preparations [7,32,33]. For example, a combination of MSCs and HSCs was suggested to produce a joint neuroprotective effect over the use of a single stem cell type [34]. The combination of MSCs and HSCs has been shown to cooperate to form a niche to promote haematopoiesis versus differentiation. In this niche, MSCs act as a feeder layer, maintaining HSCs in an undifferentiated state. When HSCs are allowed to differentiate during their expansion, they undergo cell ageing and death [34]. Previous work has shown that NC administration provides neuroprotection in models of amyotrophic lateral sclerosis, frontotemporal dementia, and spinal cord injury [7,32,33].

The goal of the current study was to investigate the potential effects of the NC preparation on the hallmarks of AD-like manifestations in a commonly used AD model, APPswe/PS1dE9 mice. We focused on gene expression changes in insulin receptor-mediated signalling *Ir*, *Igfr1*, *Irs2*, and *Igf* [24], related neurotrophic factors *Bdnf and Syp,* and mitochondrial regulator *Pgc-1α* [35], as well as markers of ageing associated with AD, *Egr1*, *Gdf15*, *Sitr1*, *Sqstm*, *Arc*, and *Cldn5* [36,37,38,39,40,41] in the prefrontal cortex of APPswe/PS1dE9 mice. These mutants express the Swedish mutation of the amyloid precursor protein (APP) and mutated PSEN1/PSEN2 genes that lead to the upregulation of β- and γ-secretases and Aβ accumulation [42], formation of tau tangles in the brain, and impaired memory and emotionality [43,44].

Twelve-month-old female APPswe/PS1dE9 mice received an injection of NC or a vehicle into the cisterna magna, and forty days thereafter were studied for memory deficits and altered emotionality compared to wild-type (WT) littermates that received a vehicle injection. The cortex, hippocampus, and thalamus were studied for amyloid plaque density (APD) of different sizes, <50 μm^2^, 50–100 μm^2^, 100–200 μm^2^, 200–500 μm^2^, and >500 μm^2^, and total APD using 6E10 immunohistochemical staining. The gene expression of selected markers was studied using RT-PCR.

## 2. Materials and Methods

### 2.1. Animals and Study Design

APPswe/PS1dE9female mice, aged 12 months, along with WT counterparts, were bred on a C57BL/6 background. The mice underwent genotyping and were housed in small groups under typical laboratory conditions, featuring a reversed 12 h light–dark cycle (lights on at 21:00), with unrestricted access to food and water in a controlled environment (22 ± 1 °C, 55% humidity). All experimental procedures adhered to the European Communities Council Directive on the care and use of laboratory animals 2010/63/EU, following approval from the Local Ethics Committee PE “National Laboratory of Astana”, Nazarbayev University, dated 20 March 2023, N02-2023, concerning animal care and welfare, and in line with ARRIVE guidelines (accessed the last time on 1 March 2025). Randomisation of the groups was carried out by balancing body weight, and no animals were excluded from the study. Experimenters were blinded to the animals’ identities in all parts of the experiments.

#### Study Flow

**Figure 1:**
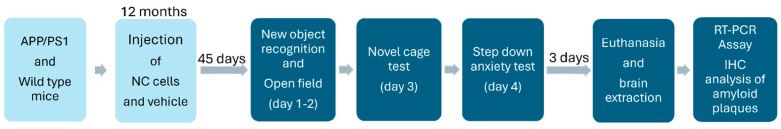
Experimental design. Twelve-month-old APPswe/PS1dE9 transgenic mice were injected with NCs into the cisterna magna. Groups of transgenic and wild-type mice received vehicle injections. Behavioural assessments were conducted over a four-day period, beginning forty-five days post-injection. Three days after the final behavioural test, the animals were euthanised, and their brains were collected for analysis. RNA was extracted for RT-PCR assay, and brain sections were processed for immunohistochemical analysis (IHC) of amyloid plaque burden.

Adjusting previously employed protocol of central administration, NC preparation (see below) was injected into the cisterna magna of APPswe/PS1dE9 (n = 10) mice; a fresh NC sample suspended in Ringer solution was used. Another subgroup of APPswe/PS1dE9 (n = 16) and WT mice (n = 19) received vehicle (Ringer solution). Randomisation was performed prior to the onset of treatment. In total, 45 mice were used. Following a forty-five-day period, all mice were examined in a battery of behavioural tests (see below), and animals were weighed weekly. The size of APPswe/PS1вE9-NC was limited by the short vitality of fresh NC and time-consuming i.c. surgery procedure (Figure 1).

During the final week of the experimental period, all animals underwent a sequence of behavioural assessments. On Days 1 and 2, the open field test and novel object recognition test were conducted. On Day 3, the novel cage test was performed, followed by the step-down anxiety test on Day 4. All tests were conducted under standardised conditions, with potential confounding factors systematically controlled by our laboratory. Three days after the final behavioural test, mice were euthanised (see below). The brain was bisected: one hemisphere was used to isolate the prefrontal cortex for RNA extraction, cDNA synthesis, and quantitative RT-PCR analysis (see below); the other hemisphere was fixed and processed for immunohistochemical analysis of amyloid plaque burden using the 6E10 antibody (see below).

No animals were excluded from analysis, and no pre-defined exclusion criteria were applied. Experimenters were blinded to group allocation until after data collection and analysis were completed.

### 2.2. Neuro-Cells Preparation

NC, a formulation comprising human bone marrow-derived HSCs and MSCs, with a composition of 1.39 × 10^6^ MSCs and HSCs, including 5 × 10^5^ CD34^+^ cells, was supplied by Neuroplast BV (Maastricht, The Netherlands). The expression profile of MSC markers was overlapping; within the total cell preparation, 85.6% were CD105^+^, 13% were CD90^+^, 7% were CD271^+^, and 4% were CD73^+^ as determined by single fluorescence-activated cell sorting (FACS) staining [33]. Following production, NC was transported under controlled conditions at 4 °C to the experimental site within 24 h and administered within the subsequent 36 h. The vitality of NC was assessed 2 h prior to administration, as described by de Munter et al. (2020) [33]. Before administration, the cells were resuspended in Ringer’s solution, as previously detailed by de Munter et al. (2020) [33].

### 2.3. Central Administration of Neuro-Cells

The administration protocol for NC was based on prior research, which demonstrated extensive distribution and tolerability of intracerebral (i.c.) administration of NC into mice [33,35]. The animals were anesthetised using halothane (Halothane TM; Willy Rusch, Boblingen, Germany) and secured in a stereotaxic frame (World Precision Instruments, Sarasota, TX, USA) for unilateral intracerebral infusion via a cranial opening in the mice, as previously described [33,35]. All Neuro-Cell preparations were derived from the same stock sample. A volume of 10 µL of either NC or Ringer solution was administered using an automated intelligent motorised stereotaxic instrument (RWD Life Science Co., Shenzhen, China) equipped with an integrated atlas, over a period of 10 min. The stereotaxic coordinates were as follows [45,46]: antero-posterior, −1.1 mm relative to the Obex; medio-lateral, +0.5 mm relative to the brainstem midline; and dorso-ventral, −1.3 mm depth relative to the brainstem surface. General anesthesia was induced with 5% isoflurane (Laboratorios Karizoo S.A., Barcelona, Spain) in oxygen and maintained with 3–4% isoflurane in oxygen. Following the injections, the needle was withdrawn, and the incision was sutured using Vicryl rapide 5.0 (Ethicon, Somerville, NJ, USA). The animals were kept in a warm environment for recovery and monitored for 24 h post-surgery, with analgesia administered for the first two days thereafter.

### 2.4. Behavioural Assays for Memory, Locomotion and Anxiety

#### 2.4.1. Open Field and Object Exploration/Recognition Paradigm

The open field test was conducted in a square enclosure (45 × 45 × 45 cm, Technosmart, Rome, Italy) with a light intensity of 5 lux. The animal was positioned in the behaviour of the box, and its movements were monitored for a duration of 5 min using a digital camera placed above the arena. The number of crossed sectors (5 × 5 cm each), time spent in the behaviour (area 15 × 15 cm in the behaviour), duration of freezing, and grooming behaviour were assessed through automated offline analysis (ViewPoint, Civrieux, France) as previously described [47]. This assay was integrated with the object exploration/recognition paradigm [47,48]. Five minutes after the animal was introduced to the arena, two identical small objects (“brush,” approximately 7 × 4 × 3 cm) were gently affixed symmetrically, 4 cm apart, on the surface of the apparatus. The latency of the first exploration of either object by the mouse was recorded. Objects used in the new object recognition test were either disposable and novel for each mouse (flowers made from paper) or reusable and washable with water and mild detergent (plastic brush), ensuring that any contamination (such as the scent of a preceding mouse) was effectively eliminated. The mouse was permitted to explore the arena and objects freely for the subsequent 15 min.

On the subsequent day, mice were assessed for novel object exploration and recognition. During this assessment, one object was substituted with a new object, referred to as the “flower” (approximately 7 × 4 × 3 cm in size). The mouse was placed equidistant from the objects within the same arena and permitted to explore freely for a duration of 15 min. The duration of object exploration was defined as the time when the mouse’s nose was oriented towards the object while positioned at a distance of less than 2 cm from it. This exploration time was recorded offline for each object, as well as for both objects combined, using VideoTrack software (ViewPoint Behavior Technology, VideoTrack 3.10, Civrieux, France). The percentage of exploration time dedicated to the “new object,” which replaced the “familiar object” from Day 1 to Day 2, was compared against a 50% chance level of approaching either object. This measure was utilised as an indicator of object recognition memory.

#### 2.4.2. Novel Cage Test

The novel cage test was conducted to evaluate vertical exploration activity in a novel environment. A mouse was placed in a transparent plastic cage (14 × 21 × 27 cm) containing a small amount of fresh litter, under a light intensity of 5 lux. The number of rearing behaviours and the latency to the first rear were recorded over a 3 min period [47,49].

#### 2.4.3. Step-Down Anxiety Test

The step-down anxiety test was employed to evaluate anxiety-like behaviour in mice by measuring the latency of stepping down, as previously described by Strekalova et al. (2018) and Vignisse et al. (2017) [50,51]. Mice were initially placed on a platform measuring 7 × 7 × 1 cm and enclosed within a cylinder with a diameter of 7 cm and a height of 15 cm. Subsequently, they were transferred to a transparent cubical apparatus measuring 30 × 30 × 50 cm (Technosmart, Rome, Italy). Upon removal of the cylinder, the latency for the mice to step down from the platform with all four paws was recorded as an indicator of anxiety.

### 2.5. Culling and Brain Tissue Collection

Mice were terminally anesthetised using isoflurane inhalation to facilitate subsequent material collection. The animals underwent transcardial perfusion with 10 mL of ice-cold saline, after which a portion of the brain containing the prefrontal cortex was isolated and stored at −80 °C for later gene expression analysis, as detailed previously [47,52]. This procedure was followed by perfusion with 4% paraformaldehyde via the left ventricle. The second half of the brain was then excised, post-fixed in formaldehyde overnight as described by Strekalova et al. 2022) [53], and subsequently embedded in paraffin for histological analysis.

### 2.6. Brain Sectioning and Histological Assays

Fixed tissue was dehydrated using rising concentrations of ethanol solutions, incubated in chloroform, and then embedded in paraffin, as described previously [54]. Paraffin sections covering a 600 μm hippocampal zone were cut at 8 μm and mounted on polylysine-coated slides (Thermo Scientific Inc., Kalamazoo, MI, USA), using Leica EG1160 tissue embedding station (Leica Biosystems, Germany), as described previously [54]. For tissue staining, the sections were deparaffinised in a xylene bath for 20 min; rehydrated through graded ethanol solutions (100% for 20 min, 95% for 5 min, and 50% for 5 min); and washed three times with deionised water for 5 min.

### 2.7. Immunohistochemical Staining of Amyloid Plaques with 6E10

**Figure 2:**
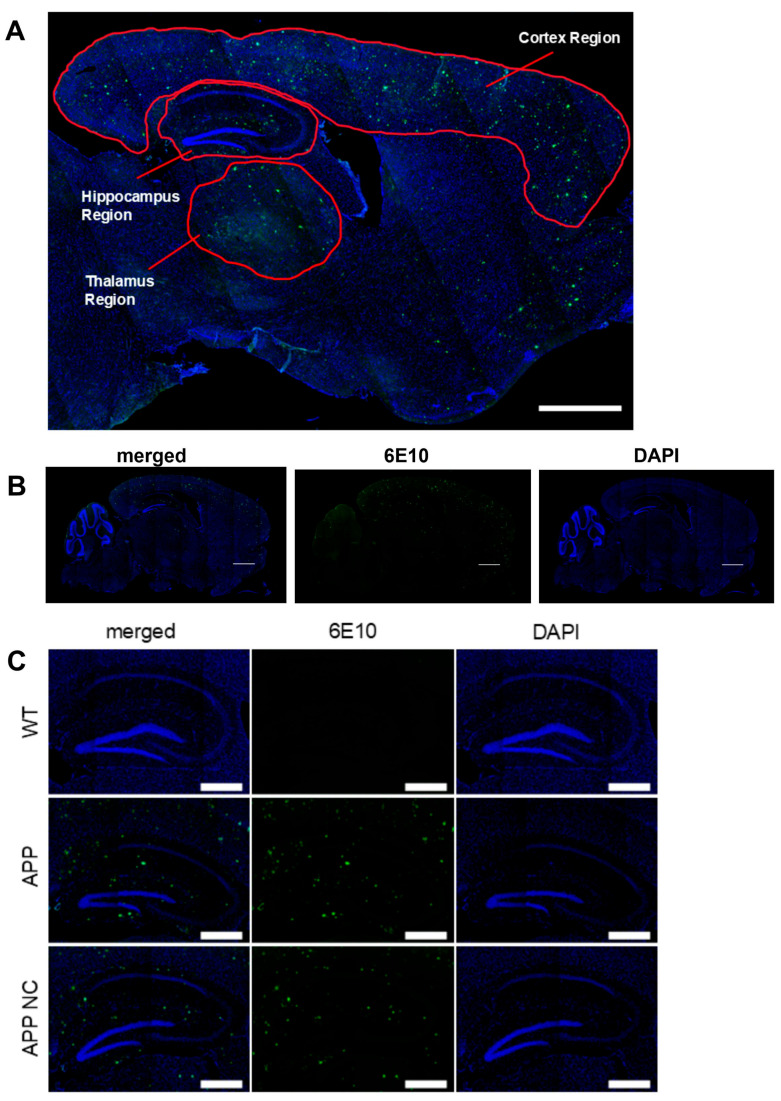
Anatomical regions of the brain investigated in the study and representative images of IHC staining. (**A**) Brain areas of interest: cortex, hippocampus, and thalamus examined in APPswe/PS1dE9 mice, at ×10 magnification, scale bar—800 µm. (**B**) Representative images of anti-amyloid IHC staining (6E10 antibody) and DAPI, x10 magnification, scale bar—1 mm. (**C**) Images of 6E10, DAPI, and 6E10+DAPI staining in the brains of APPswe/PS1dE9 mice, ×10 magnification, scale bar—1 mm.

**Figure 3:**
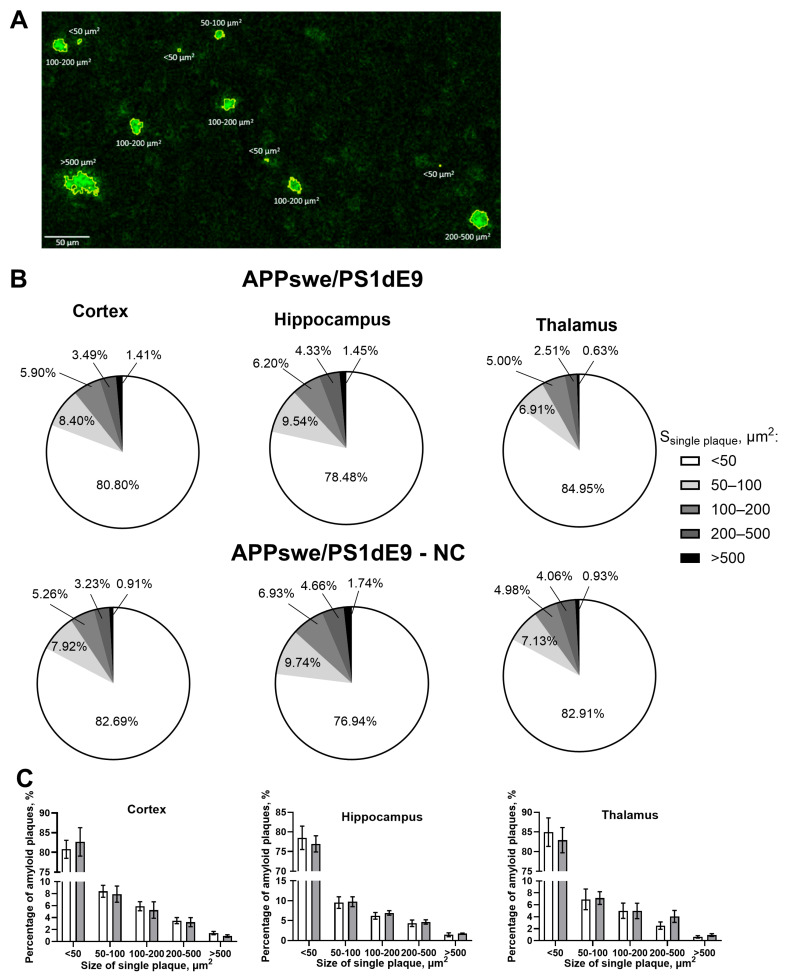
Plaque size distribution across cortex, hippocampus, and thalamus in untreated APPswe/PS1dE9and NC-APPswe/PS1dE9 mice. (**A**) The 6E10-stained amyloid plaques of various size ranges: <50 μm^2^, 50–100 μm^2^, 100–200 μm^2^, 200–500 μm^2^, and >500 μm^2^, scale bar—50 µm. (**B**) Similar proportions of various plaque sizes in the cortex, hippocampus, and thalamus in untreated APPswe/PS1dE9 and NC-APPswe/PS1dE9 mice. (**C**) The percentage of 6E10-stained amyloid plaques of various sizes did not differ significantly between untreated APPswe/PS1dE9 mice (white bars) and NC-APPswe/PS1dE9 group (grey bars), two-way ANOVA, and Šídák’s test; scale bar—50 µm. Data are presented as mean ± SEM (n = 4).

Amyloid plaques were determined with the 6E10 antibody (Figure 2B and Figure 3A). Immunostaining was performed overnight using primary anti-beta-amyloid antibody (mouse monoclonal antibody, anti-β-Amyloid, 1–16 Antibody (Clone 6E10), SIG-39320, BioLegend Inc., San-Diego, CA, USA, diluted 1:1000), followed by 2 h long incubation with goat anti-mouse IgG (H+L) Highly Cross-Absorbed Antibodies (A28175, Alexa Fluor™ 488, Invitogen™, Thermo Fisher Scientific Inc., Carlsbad, CA, USA, diluted 1:1000) at room temperature. Nuclei were stained with DAPI (62248, Thermo Fisher Scientific Inc., Carlsbad, CA, USA, diluted 1:1000) for 5 min and embedded with the Epredia™ Immu-Mount™ water-based mounting medium (Thermo Fisher Scientific Inc., Kalamazoo, MI, USA). A total of 5 slices per animal was analysed using confocal laser scanning microscopy LSM880 in the tile scan mode (Carl Zeiss, Oberkochen, Germany), as described elsewhere [54,55] (Figure 2A). Image processing of β-amyloid deposits’ morphometric analysis was based on QuPath 0.4.3. pixel classifier (Belfast, Northern Ireland, UK, [56,57]), a machine learning algorithm for the detection of three examined areas (Figure 2A): the hippocampal region, thalamus region, and cortex region [54,55]. The size of the quantified area was 400 um^2^ for each anatomic region. Deep learning analysis was used to determine plaques of various sizes, <50 μm^2^, 50–100 μm^2^, 100–200 μm^2^, 200–500 μm^2^, and >500 μm^2^, as described previously [54]. The number of each type of plaque was calculated per mm^2^ in each of the examined brain regions, and the total amyloid plaque density (APD) was calculated; the total APD for all types of plaques was calculated for cortex, hippocampus, and thalamus. Since WT mice do not develop detectable amyloid plaques, their inclusion in the quantitative comparison of plaque burden in APPswe/PSd1E9-treated mice would not be meaningful and would distort the statistical analysis.

### 2.8. QuPath 0.4.3 Pixel Classifier

QuPath (URL https://qupath.github.io accessed on 6 March 2025) was used [56,57] to annotate and classify Aβ plaques in immunohistochemistry images, employing pixel classifiers and region-of-interest analyses to quantify pathology across brain regions [58,59].

### 2.9. Real-Time Polymerase Chain Reaction (RT-PCR)

RNA was extracted from tissue using the QIAzol Lysis Reagent and the RNeasy Mini Kit (QIAGEN Sciences Inc., Germantown, MD, USA). Subsequently, first-strand cDNA synthesis was conducted utilising random primers and the QuantiTect Reverse Transcription Kit (QIAGEN Sciences Inc., Germantown, MD, USA), wherein 1 μg of total RNA was converted into cDNA. Quantitative reverse transcription PCR (RT-PCR) was executed using the SYBR Green Master Mix (Bio-Rad Laboratories, Philadelphia, PA, USA). The qRT-PCR was performed in a 10 μL reaction volume, comprising 5 μL of SYBR Green Master Mix, 3 μL of RNase-free water, 1 μL of specific forward and reverse primers at a concentration of 20 pmol/μL, and 1 μL of cDNA. The initial denaturation step for qRT-PCR was conducted at 95 °C for 5 min, followed by 40 cycles of denaturation at 95 °C for 30 s and annealing at 60 °C for 30 s. The sequences of the primers employed are detailed in Table S1 (see the Supplementary File); all primers were procured from Life Technologies (Thermo Fisher Scientific Inc., Carlsbad, CA, USA). All samples were analysed in triplicate as described in previous studies [7,46].

### 2.10. Statistical Analysis

Data were analysed utilising GraphPad Prism version 9.1.0 (San Diego, CA, USA). The Shapiro–Wilk test was employed to assess normality. For data exhibiting a normal distribution, comparisons among three groups were conducted using ordinary one-way ANOVA, followed by Holm–Šídák’s test when variances were equal as determined by Bartlett’s test; otherwise, Welch’s ANOVA with post hoc Dunnett’s T3 test was applied. Two-way ANOVA followed by Tukey’s post hoc test was used for comparisons of four groups. For data not normally distributed, the Kruskal–Wallis test with post hoc Dunn’s test was used for three-group comparisons. For two-group comparisons with a normal distribution, unpaired Welch’s *t*-tests were utilised. For comparisons with random levels, a one-sample *t*-test was applied, given normally distributed data. In our investigation to validate the APPswe/PS1dE9 model and evaluate the efficacy of NC in APPswe/PS1dE9 mice, group comparisons were restricted to those differing by a single factor, namely, WT versus non-treated APP/PS1, and untreated APPswe/PS1dE9 versus APPswe/PS1dE9 group. The significance level was established at 95% (*p* < 0.05). No datapoints were excluded from analysis. Data were presented as Mean ± SEM, and data not normally distributed were presented as Median or Median with interquartile range.

## 3. Results

### 3.1. Injection of NC to APPswe/PS1dE9 Mice Reduces Amyloid Plaque Formation of the Smallest Size

The results of amyloid plaque size distribution across cortex, hippocampus, and thalamus in untreated APPswe/PS1dE9 and NC-APPswe/PS1dE9 mice revealed similar proportions of various plaque sizes in the two groups (Figure 3A–C).

**Figure 4:**
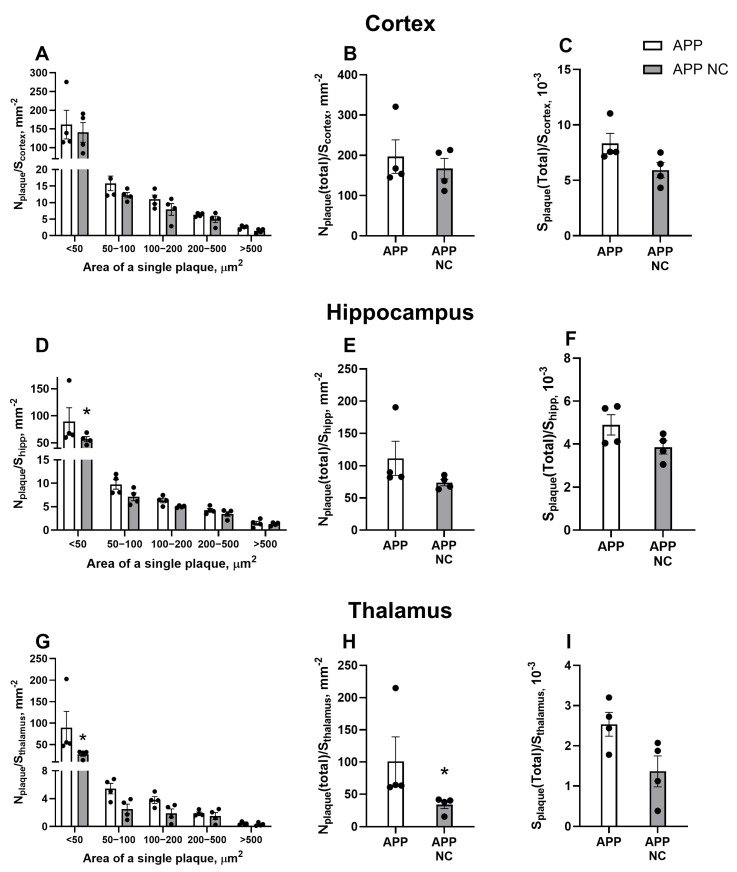
Density of amyloid plaques of varying sizes in different brain regions of APP/PS1 mice following treatment with Neuro-Cells. In the cortex, no statistically significant differences were observed in (**A**) the density of plaques of different sizes, (**B**) total plaque density, and (**C**) total plaque area between untreated APPswe/PS1dE9 and APPswe/PS1вE9-NC mice. In the hippocampus, as determined by two-way ANOVA followed by Šídák’s test, a significant reduction in (**D**) the density of the smallest plaques (<50 μm^2^) was observed in to APPswe/PS1вE9-NC mice, while no significant differences were found in the (**E**) total plaque density and (**F**) the total plaque area between the groups. Similarly, in the thalamus, a significant decrease in (**G**) the density of the smallest (<50 μm^2^) plaques was observed in the APPswe/PS1вE9-NC group. Moreover, the Mann–Whitney test revealed that in the thalamus (**H**), the total plaque density was significantly lower in APPswe/PS1вE9-NC animals, but no significant difference was found in (**I**) the total plaque area. WT = wild-type controls, APP = APPswe/PS1dE9 mice, APP NC = APPswe/PS1dE9 mice treated with NC. Data are presented as mean ± SEM *(n* = 4). Statistical significance was assessed using two-way ANOVA followed by Šídák’s multiple comparisons test for panels (**A**,**D**,**G**), and the Mann–Whitney test for panels (**B**,**C**,**E**,**F**,**H**,**I**). * *p* < 0.05.

APD revealed no significant differences in the density of plaques of various sizes (<50 µm^2^: *p* = 0.8631, 50–100 µm^2^: *p* > 0.9999, 100–200 µm^2^: *p* > 0.9999, 200–500 µm^2^: *p* > 0.9999, >500 µm^2^: *p* > 0.9999, two-way ANOVA and Šídák’s test), total plaque density (*p* = 0.6857, Mann–Whitney test), or plaque area (*p* = 0.0571, Mann–Whitney test) in the cortex of untreated APPswe/PS1dE9 mice and those treated with NCs (Figure 4A–C). However, the two-way ANOVA test showed a significant reduction in the density of small plaques (<50 µm^2^) in the hippocampus of NC-treated APPswe/PS1dE9 mice compared to untreated mice (*p* = 0.0421; Figure 4D). No significant differences were found in the density of larger plaques (50–100 µm^2^: *p* =0.9998, 100–200 µm^2^: *p* > 0.9999, 200–500 µm^2^: *p* > 0.9999, >500 µm^2^: *p* > 0.9999), total plaque density (*p* = 0.1143), or plaque area in the hippocampus (*p* = 0.3429) between the groups (Figure 4E,F). A similar effect was observed in the thalamus, where NC treatment led to a significant reduction in the density of small plaques (<50 µm^2^) (*p* = 0.0054; Figure 4G), while the density of larger plaques remained unchanged. In addition, the Mann–Whitney test revealed a significant decrease in total plaque density in the thalamus of NC-treated to APPswe/PS1dE9 mice (*p* = 0.0286; Figure 4H). However, no significant differences in total plaque area were detected between the groups (*p* = 0.1143).

### 3.2. Administration of NC Ameliorates Hippocampus-Dependent Learning and Anxiety-like Behaviour in APPswe/PS1вE9-NC Mice

**Figure 5:**
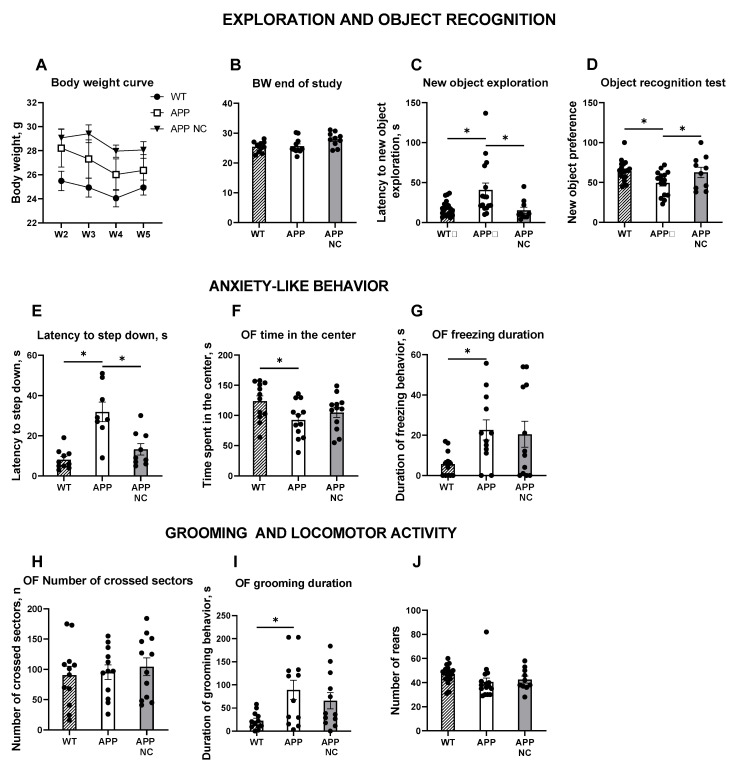
Effects of NC treatment on object recognition learning and anxiety-like behaviour. (**A**) Body weight changes over the 5-week experimental period. (**B**) Body weight of the mice at the start of the behavioural study. (**C**) Latency to explore the novel object was significantly increased in untreated APPswe/PS1dE9 mice, indicating neophobic behaviour. NC-treated mutants had reduced the latency of the latency to approach an object that was close to the values observed in WT mice. (**D**) Analysis of new object recognition memory revealed that APPswe/PS1dE9 mice had no significant preference for a novel object, which was also significantly lower than in NC-treated mutants. (**E**) The scoring of latency to step down showed that APPswe/PS1dE9 mice exhibited significantly longer latency compared to WT animals. NC treatment significantly reduced the latency in APPswe/PS1dE9 mice. (**F**) In the open field test, APPswe/PS1dE9 mice spent significantly less time in the behaviour, indicative of increased anxiety-like behaviour. NC-treated APP/PS1 mice exhibited a duration of time spent in the behaviour of the open field that was close to values of the WT group (**H**). The number of crossed sectors was not significantly different between the groups. (**I**) The duration of grooming behaviour was significantly longer in untreated APPswe/PS1dE9 mice compared to WT controls. However, in NC-treated transgenic mice, no significant differences were observed compared to neither WT nor untreated APPswe/PS1dE9 animals. (**J**) No statistically significant differences in the number of rearing events were observed between the groups. WT = wild-type controls, APP = APPswe/PS1dE9 mice, APP NC = APPswe/PS1dE9 mice treated with NC. Average group sizes: WT, n = 14; APP, n = 13; APP NC, n = 11. Statistical significance was assessed using two-way ANOVA and Tukey’s post hoc test for panel A; one-way ANOVA and Tukey’s post hoc test for panels (**B**,**F**–**J**); Kruskal–Wallis test and Dunn’s post hoc correction for panel C; one-way ANOVA and Holm–Šídák’s post hoc test for panel D; Brown–Forsythe ANOVA and Dunnett’s T3 post hoc test for panel E. * *p* < 0.05. Data are presented as mean ± SEM.

No significant differences in body weight were observed between the groups over the experimental period, while APPswe/PS1вE9-NC mice exhibited transient reduction in body weight during weeks 2 to 5 of treatment (*p* = 0.011 for W2; *p* = 0.0016 for W3; *p* = 0.0011 for W4; *p* = 0.0087 for W5; one-way ANOVA and Tukey’s post hoc test; Figure 5A) that was not longer observed by the start of behavioural studies (APP: *p =* 0.9672, APP NC: *p* = 0.0519 compared to the WT, Figure 5B). The novel object recognition test revealed significantly longer latency in APPswe/PS1dE9 group to approach the novel object compared to WT animals (*p* = 0.0246, Kruskal–Wallis test and Dunn’s post hoc correction), suggesting neophobic behaviour of the mutants. Notably, NC-treated mutant mice showed a significant reduction in this measure compared to untreated transgenic animals, with the values that were close to control measurements (*p* = 0.0073; Figure 5C).

In the novel object recognition test, untreated APPswe/PS1dE9 mice showed no preference for a novel object on a recall session, indicating impaired object recognition memory (*p* = 0.0376, one-sample *t*-test; Figure 5C). Preference for a new object was significantly lower in the untreated mutants than in WT animals (*p* = 0.0181), but not in NC-injected APPswe/PS1dE9 mice (*p* = 0.0439; Figure 5D).

In the step down anxiety test, the latency to step down of untreated APPswe/PS1dE9 mice was significantly longer than in WT animals exhibited (*p* = 0.0033, Brown–Forsythe ANOVA and Dunnett’s T3 post hoc test; Figure 5E), while mutants treated with NC did not exhibit such change (*p* = 0.2683), showing a significant reduction as compared with untreated transgenic mice (*p* = 0.0138; Figure 5E). These data support a finding of elevated anxiety in untreated APPswe/PS1dE9 mice.

Furthermore, in the open field test, untreated APPswe/PS1dE9 mice spent significantly less time in the center of the apparatus compared to WT mice (*p* = 0.0449; Figure 5F), indicating increased anxiety-like behaviour. NC-treated mutant mice showed no significant differences in this parameter compared to either WT or untreated transgenic mice (*p* = 0.3032, *p* = 0582, respectively; Figure 5F). Analysis of freezing duration revealed a significant increase in APPswe/PS1dE9 mice compared to WT controls (*p* = 0.0479; Figure 5G). However, NC-treated transgenic mice showed no statistically significant differences in freezing behaviour compared to either WT or untreated APPswe/PS1dE9 mice (*p* = 0.0935, *p* = 0.9472, respectively; Figure 5G), suggesting no measurable effect of the treatment on fear-related responses. We found no significant differences in the number of crossed sectors between the groups (APP: *p* = 0.9669, APP NC: *p* = 0.7607, compared to the WT, APP NC: *p* = 0.8901, compared to the APP; Figure 5H), indicating unaltered general locomotion of experimental groups of mice. The duration of grooming behaviour was significantly increased in untreated APPswe/PS1dE9 mice compared to WT controls (*p* = 0.0162), which manifested signs of stress-related behaviour in these animals. NC-treated mice showed no significant changes in grooming behaviour (*p* = 0.1531, compared to the WT, *p* = 0.5645, compared to the APP; Figure 5I). In the novel cage test, there were no significant differences between the groups in the number of rears revealed that showed their unaltered vertical activity (APP: *p* = 0.1373, APP NC: *p* = 0.4859, compared to the WT, APP NC: *p* = 0.8610, compared to the APP; Figure 5J).

### 3.3. Gene Expression of Bdnf, Elements of IR-Mediated Signalling, and Markers of Ageing and AD in APPswe/PS1вE9-NC Mice

**Figure 6:**
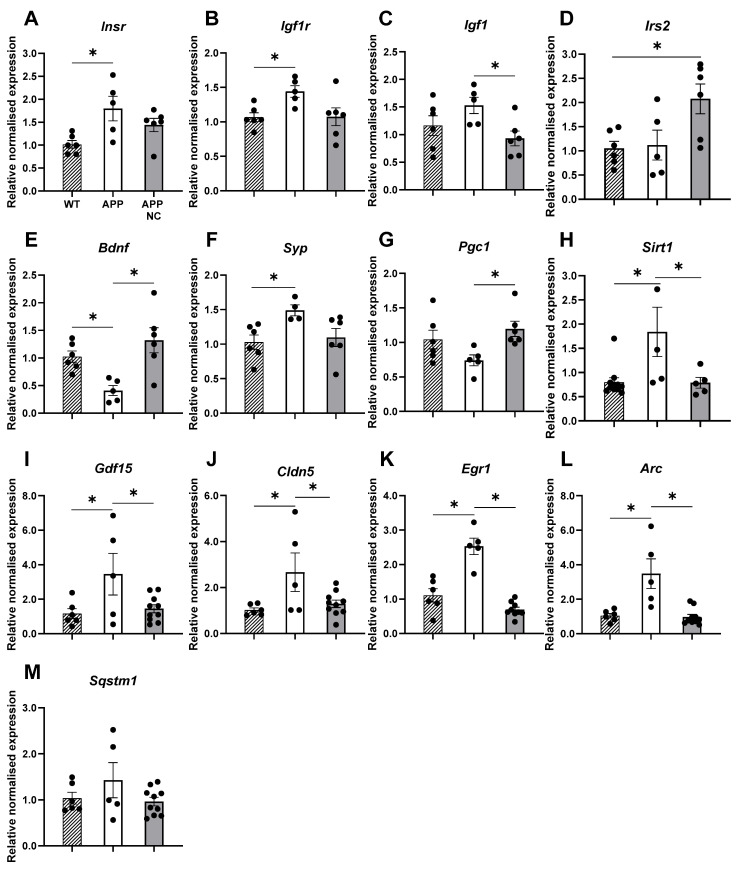
Expression of IR-related genes in WT mice, untreated APPswe/PS1dE9 mice, and treated with NC. (**A**) The expression of *Insr* was increased in APPswe/PS1вE9 mice compared to WT (*p* = 0.0162). (**B**) The expression of *Igf1r* was elevated in APPswe/PS1dE9 mice in comparison with controls. (**C**) No significant differences in *Igf1* expression were observed between untreated APPswe/PS1dE9 and WT mice, while NC-treated mutants had reduced *Igf1* expression in comparison with mutants (*p* = 0.0463). (**D**) NC-treated mutant animals had significantly increased *Irs2* expression (*p* = 0.0313). (**E**) Cortical *Bdnf* expression was significantly decreased in APPswe/PS1dE9 mice (*p* = 0.0451) and increased in NC-treated mutants, in comparison to control levels (*p* = 0.0038). (**F**) The expression of *Syp* was significantly increased in untreated APPswe/PS1dE9 mice compared to WT controls (*p* = 0.0448). In the NC-treated group, *Syp* expression did not differ significantly from either WT or untreated APPswe/PS1dE9 mice. (**G**) NC-treated mutants showed significantly increased *Pcg-1a* expression compared to untreated transgenic animals. (**H**) *Sitr1* expression was significantly higher in untreated APPswe/PS1dE9 mice compared to WT controls. NC treatment significantly reduced *Sitr1* expression in APPswe/PS1dE9 mice. (**I**–**L**) The expression level of *Gdf15*, *Cldn5*, *Egr1*, *and Arc* genes was significantly higher in untreated APPswe/PS1dE9 mice, whereas no such changes were found in NC-treated mutants. (**M**) Cortical *Sqstm1* expression did not differ significantly between the groups. WT = wild-type controls, APP = APPswe/PS1dE9 mice, APP NC = APPswe/PS1dE9 mice treated with NC. Data are presented as mean ± SEM (average group sizes: WT, n = 6; APP, n = 5; APP NC, n = 7). Statistical significance was assessed using one-way ANOVA followed by Tukey’s post hoc test. * *p* < 0.05.

Gene expression changes described in this section are also shown in Table 1 and Supplementary Figure S1. One-way ANOVA and Tukey’s post hoc test revealed a significantly elevated expression of *Insr* in the cortex of untreated APPswe/PS1dE9 mice compared to WT controls (*p* = 0.0162; Figure 6A). However, no significant change was observed in *Insr* expression in NC-treated APPswe/PS1dE9 mice (*p* = 0.1956, *p* = 0.3371, compared to WT and APP, respectively; Figure 6A). Analysis of *Igf1r* expression showed a significant increase in the cortex of untreated APPswe/PS1dE9 mice (*p* = 0.0483; Figure 6B). In transgenic mice treated with NC, cortical *Igf1r* expression remained at the WT level (Figure 6B). The cortical expression level of the *Igf1* gene in untreated APPswe/PS1dE9 mice did not differ significantly from WT mice. However, in the cortex, NC treatment of APPswe/PS1dE9 mice resulted in a significant decrease in *Igf1* expression compared to untreated APP/PS1 mice (*p* = 0.0463; Figure 6C).

The analysis of *Irs2* gene expression demonstrated no significant difference between the groups (APP: *p* = 0.981, compared to control, APP NC: *p* = 0.0568; Figure 6D). However, NC treatment led to a significant increase in *Irs2* expression in APPswe/PS1dE9 mice compared to WT controls (*p* = 0.0313). The *Bdnf* expression in untreated APPswe/PS1dE9 mice was significantly lower compared to WT animals (*p* = 0.045; Figure 6E). Moreover, NC treatment significantly increased *Bdnf* expression in APPswe/PS1dE9 mice, rescuing this parameter to near-control levels (*p* = 0.0038; Figure 6E). *Syp* expression revealed a significant increase in untreated APPswe/PS1dE9 mice compared to WT controls (*p* = 0.0448; Figure 6F). In NC-treated APPswe/PS1dE9 mice, the expression of this gene did not differ significantly from that of WT animals (*p* = 0.8966; Figure 6F).

*Pcg-1α* expression showed no significant difference between APPswe/PS1dE9 and WT mice (*p* = 0.1991; Figure 6G). However, NC treatment resulted in a significant increase in *Pcg* expression in APPswe/PS1dE9 mice compared to untreated transgenic animals (*p* = 0.0387; Figure 6G).

In untreated APPswe/PS1dE9 mice, the *Sitr1* expression was significantly higher compared to WT controls (*p* = 0.0124). NC treatment significantly reduced *Sitr1* expression in APPswe/PS1dE9 mice (*p* = 0.0316; Figure 6H). The results of the cortical *Gdf15*, *Cldn5*, *Egr1* and *Arc* gene expression assay showed a similar pattern (Figure 6I–K): the expression level of these genes was significantly higher in untreated APPswe/PS1dE9 mice compared to WT animals (*p* = 0.0408, *p* = 0.0295, *p* < 0.0001, *p* = 0.018, respectively). NC treatment significantly reduced *Gdf15*, *Cldn5*, *Egr1* and *Arc* expression in APPswe/PS1dE9 mice in comparison to untreated mutants (*p* = 0.0485, *p* = 0.0456, *p* < 0.0001, *p* = 0.0005, respectively; Figure 6I–K).

No statistically significant differences in cortical *Sqstm1* expression were observed among the experimental groups (APP: *p* = 0.3926, APP NC: *p* = 0.9526, compared to the WT, APP NC: *p* = 0.2108, compared to the APP; Figure 6M).

## 4. Discussion

Our study demonstrated that the administration of NC, a new stem cell preparation, ameliorated experimental AD-like syndrome. A single i.c. infusion of NC to APPswe/PSd1E9 mutants significantly counteracted the accumulation of amyloid plaques of the smallest size in the hippocampus and thalamus, rescued hippocampus-dependent memory, and prevented excessive grooming and anxiety-like changes in the open field and step-down anxiety test without altering body weight and general locomotion. The amelioration of the hallmarks of AD was associated with increased expression of *BDNF, Irs2,* and *Pgc-1α*, overly normalised gene expression of neurotrophin- and IR-related molecules, and established markers of ageing. These findings are in keeping with previously published results demonstrating the beneficial action of bone marrow-derived stem cell preparations on the manifestations of neurodegeneration [15,16,17,18,60,61,62,63] and evidence of neuroprotective effects of NC obtained in rat and mouse models of neurological disorders [7,32,33,36].

Specifically, we demonstrated elevated *Bdnf* expression in the cortex of NC-treated APPswe/PSd1E9 mice, which is consistent with previous reports showing elevated BDNF levels following the administration of MSCs associated with increased hippocampal neurogenesis and an upregulation of neuronal synaptic plasticity, learning, and memory [14,15,16]. Furthermore, transplanted bone marrow-derived stem cells have previously been shown to secrete BDNF along with other neurotrophins, such as IGF-1, HGF, and NGF [17,18]. MSCs administration also boosted the expression of several molecules with neurotrophic functions, such as VEGF [12,64], doublecortin, Ki-67, and nestin [15,16]. BDNF and other neurotrophins have been shown to counteract the damaging action of inflammation and oxidative stress in AD neurons and to decrease amyloid plaque formation. As such, the stimulatory effect of NC on *Bdnf* expression reported in our study is likely to underlie the effects of NC on amyloid accumulation and the behaviour of APPswe/PSd1E9 mice.

In the untreated APPswe/PSd1E9 mice, we found an increase in *Syp* gene expression, which is in contrast with clinical evidence in patients with AD [38,65]. This increase, however, was abolished by the NC administration, suggesting that changes in untreated mice were likely compensatory and NC exerted a normalising effect on SYP functions. Similarly, increases in the expression of genes encoding IR, IGF-1R, and IGF found in untreated APPswe/PSd1E9 mice were likely compensatory and manifested insufficiency of IR-mediated signalling in these animals. Notably, these changes were not observed in the NC-treated group of mutants, which allowed us to interpret this finding as a manifestation of the beneficial effects of NC on AD pathology. Interestingly, *Irs2* was strongly upregulated in APPswe/PSd1E9-NC mice, whereas its expression was unaltered in untreated mutants. IRS2 plays an important role in the regulation of central IR-mediated signalling [22].

Bathina and Das (2015) reported that the loss or dysfunction of IRS-2 impairs neuronal survival and synaptic plasticity [66]. Tumminia et al. (2018) and Akhtar and Sah (2020) further noted that impaired IRS-2 signalling is associated with Aβ accumulation and tau hyperphosphorylation in AD [67,68]. Altered IRS-2 interferes with BDNF signalling and regulates activation of the PI3K/Akt pathway, which is critical for neuronal maintenance, contributing to AD pathology [68]. Hence, NC-induced upregulation of *Irs-2* might be another cellular mechanism of action of NC treatment underlying its positive effects on histological and behavioural changes in APPswe/PSd1E9 mice.

Consistent with previous reports of mitochondrial dysfunction in AD, typically reflected by reduced expression of PGC-1α in the brain [34], we observed a similar downregulation of *Pgc1-α* in untreated APPswe/PS1dE9 mice. In contrast, NC-treated mice exhibited normalised *Pgc1-α* expression. Given that PGC-1α has been shown to alleviate insulin resistance and oxidative stress, as well as to reduce amyloid plaque accumulation [34,37], its upregulation may underlie, at least in part, the reduction in amyloid plaque burden observed in NC-treated animals.

The untreated APPswe/PS1dE9 mice exhibited significant upregulation of several genes associated with ageing and AD-related neurodegeneration, including *Sirt1*, *Egr1*, *Gdf15*, and *Cldn5.* In contrast, the expression levels of these genes remained unaltered in NC-treated mutants. *Sirt1* plays a key role in regulating IGF-signalling, mitochondrial function, and blood–brain barrier (BBB) permeability, and is implicated in the pathophysiology of AD [36]. The immediate early gene *Egr1*, which is also upregulated in AD, has been shown to promote Aβ production and impair cholinergic signalling [40,69]. In our study, *Egr1* expression was elevated in untreated APPswe/PS1dE9 mice but remained at control levels following NC treatment.

In untreated APPswe/PS1dE9 mice, we observed significant overexpression of *Gdf15*, a gene known to increase sharply with ageing and AD, and implicated in mitochondrial dysfunction and oxidative stress [37]. This upregulation was absent in NC-treated animals, suggesting a normalising effect of the intervention. Similarly, *Cldn5*—which encodes claudin-5, a key regulator of blood–brain barrier (BBB) integrity and contributor to Aβ metabolism—is typically downregulated in ageing and AD [41,70]. However, in our study, *Cldn5* expression was elevated in untreated transgenic mice and restored to baseline levels following NC treatment. This apparent discrepancy with clinical findings may reflect differences in biological age: 12-month-old mice, while showing AD-like pathology, do not represent the advanced age typical of human post-mortem AD brain samples. It is plausible that at earlier stages of disease, certain genes associated with ageing and degeneration may exhibit compensatory upregulation, which later declines as pathology progresses.

In NC-treated APPswe/PS1dE9 mice, *Arc* expression was significantly reduced compared to untreated transgenic controls. Given that *Arc* regulates synaptic plasticity and contributes to Aβ metabolism [71,72], this downregulation may reflect a beneficial adjustment in synaptic activity, potentially contributing to the observed improvements in amyloid plaque burden and behavioural outcomes.

Although expression of *Sqstm1*, which encodes the autophagy- and oxidative stress-related protein p62 [73], did not significantly differ between untreated and NC-treated mice, a trend towards increased expression was observed in untreated mutants but not in the NC group, suggesting possible subtle effects on autophagy regulation.

These molecular changes align with prior findings showing normalisation of pro-inflammatory cytokines, including IL-1β and IL-6, in the CNS of NC-treated animals [7,32,33,35]. Such anti-inflammatory effects are consistent with known interactions between IR signalling and neuroinflammation in AD [22].

The upregulated expression of *Insr* and *Igf1r* observed in untreated APPswe/PS1dE9 mice may indicate a compensatory response to disrupted downstream signalling or reduced availability of insulin and IGF-1 protein in the brain. Notably, central insulin levels decline with age and in sporadic AD [24,74], particularly in individuals carrying the APOE ε4 allele [75]. Supporting this, in vitro studies have shown that Aβ_1–42_ can suppress insulin expression and reduce insulin levels in cultured astrocytes [76], further linking Aβ pathology to deficits in insulin signalling.

Taken together, our findings suggest that the beneficial effects of NC treatment on APD and behaviour are at least partially mediated through restoration of neurotrophin and IR-dependent signalling pathways. These effects likely include enhanced trophic support, improved mitochondrial function, and attenuation of inflammation and oxidative stress [77,78], ultimately contributing to the observed reduction in amyloid burden in NC-treated APPswe/PS1dE9 mice. Consistent with available literature linking amyloid accumulation to reduced neurotrophin levels, mitochondrial dysfunction, and cognitive and emotional deficits [77,79,80], we observed a significant reduction in the density of small plaques (<50 μm^2^) in the hippocampus and thalamus of NC-treated animals, with a similar trend in the cortex. Interestingly, these effects were most pronounced for the smallest plaques, though a comparable downward trend for plaques in the 50–100 μm^2^ range was observed across all three brain regions. Given the six-week interval between NC administration and tissue collection, which is sufficient time for new plaque formation [81], these findings suggest that NC therapy may primarily inhibit the formation of new plaques, rather than promoting clearance of existing deposits.

NC-treated APPswe/PS1dE9 mice exhibited improved performance in object recognition memory and reduced anxiety-like behaviour compared to untreated transgenic controls. These behavioural improvements are likely underpinned by the observed reduction in amyloid plaque burden and normalisation of key molecular markers, including increased *Bdnf* expression, upregulation of the mitochondrial regulator *Irs2*, and modulation of insulin receptor-related pathways. Prior studies have highlighted the close link between behavioural impairments and the underlying molecular and histopathological hallmarks of AD [82,83,84,85].

In our study, untreated APPswe/PS1dE9 mice failed to discriminate between familiar and novel objects, which is indicative of impaired hippocampus-dependent memory [86], whereas NC-treated mice showed a clear preference for novelty, suggesting preserved memory function. In addition, NC treatment mitigated anxiety-like behaviours commonly seen in AD models. Untreated APPswe/PS1dE9 mice exhibited signs of elevated anxiety, including increased latencies to step down and explore novel objects, reduced time spent in the centre of the open field, and excessive grooming—an established indicator of stress in rodents [87,88]. These behaviours were not observed in NC-treated mice, supporting the interpretation that NC normalised affective function. Importantly, no differences were detected in general locomotor activity (horizontal or vertical) between groups, indicating that behavioural effects were not confounded by changes in activity levels.

## 5. Conclusions

In summary, our findings demonstrate that treatment with NC, a novel combination of unmanipulated bone marrow-derived MSCs and HSCs, ameliorates key pathological and behavioural features in the APPswe/PS1dE9 mouse model of AD. NC administration reduced the density of small amyloid plaques, particularly in the hippocampus and thalamus, and normalised behavioural deficits in memory and anxiety. These effects were accompanied by upregulation of *Bdnf*, *Irs2*, and *Pgc1-α,* as well as restoration of insulin receptor-related signalling and expression of genes associated with ageing and neurodegeneration. As the study focused on transcriptional changes, which often precede and predict downstream protein-level effects, future work should include protein validation to strengthen mechanistic insights. Nevertheless, our results suggest that NC exerts its therapeutic effects through modulation of neurotrophin and insulin receptor pathways, enhancement of mitochondrial function, and attenuation of inflammatory and oxidative stress responses. These findings support the therapeutic potential of NC in AD and underscore the broader relevance of targeting neurotrophin- and IR-mediated signalling cascades in the development of disease-modifying treatments for neurodegenerative disorders [89,90].

## Figures and Tables

**Table 1:** Relative normalised brain gene expression in the brains of APP/PS1 and WT mice. Increased (↑) and decreased (↓) gene expression levels are indicated by arrows. Statistical significance was assessed using one-way ANOVA followed by Tukey’s post hoc test; * *p* < 0.05 vs. WT mice, ^●^ *p* < 0.05 vs. untreated APP/PS1 mice.

Gene	Relative Normalised Expression		
	WT	APP	APP NC
*Insr*	1.02 ± 0.08	↑1.80 ± 0.27 *	↑1.44 ± 0.15
*Igf1r*	1.78 ± 0.06	↑1.44 ± 0.08 *	↓1.08 ± 0.13
*Igf1*	1.17 ± 0.18	↑1.53 ± 0.15	↓0.93 ± 0.13 ^●^
*Irs2*	1.05 ± 0.15	1.12 ± 0.31	↑2.08 ± 0.33 *
*Bdnf*	1.02 ± 0.10	↓0.41 ± 0.09 *	↑1.32 ± 0.23 **^●^**
*Syp*	1.03 ± 0.11	↑1.49 ± 0.08 *	↓1.10 ± 0.13
*Pcg*	1.04 ± 0.14	↓0.74 ± 0.08	↑1.20 ± 0.11 **^●^**
*Sirt1*	0.80 ± 0.09	↑1.84 ± 0.51 *	↓0.79 ± 0.11 **^●^**
*Gdf15*	1.16 ± 0.29	↑3.46 ± 1.21 *	↓1.45 ± 0.23 **^●^**
*Cldn5*	1.02 ± 0.09	↑2.67 ± 0.84 *	↓1.29 ± 0.16 **^●^**
*Egr1*	1.11 ± 0.19	↑2.53 ± 0.24 *	↓0.70 ± 0.07 **^●^**
*Arc*	1.04 ± 0.13	↑3.48 ± 0.86 *	↓0.97 ± 0.15 **^●^**
*Sqstm1*	1.04 ± 0.13	↑1.47 ± 0.38	↓0.96 ± 0.09

## Data Availability

Data available on reasonable request. To access data, Dr. Tatyana Strekalova (t.strekalova@pharm.ox.ac.uk and tatslova@gmail.com) should be contacted.

## Supplementary Materials

The following supporting information can be downloaded at: https://www.mdpi.com/article/10.3390/cells14161293/s1, Figure S1. Heat map representation of the expression of significantly altered genes in the prefrontal cortex of WT, APP/PS1 and APP/PS1-NC mice (see ms text and Figure 5). Table S1. List of primers for RT-qPCR.

## Abbreviations

The following abbreviations are used in this manuscript:

AD	Alzheimer’s disease
MSCs	Mesenchymal stem cells
HSCs	Haematopoietic stem cells
Aβ	Amyloid-β
TNF	Tumor necrosis factor
IL-10	Interleukin-10
VEGF	Vascular endothelial growth factor
Ki-67	Marker of proliferation Kiel 67
Wnt	Wingless/Int-1
BDNF	Brain-derived neurotrophic factor
IGF-1	Insulin-like growth factor-1
HGF	Hepatocyte growth factor
NGF	Neuronal growth factor
IR	Insulin receptor
TrkB	Tyrosine receptor kinase B
IGF-1R	Insulin-like growth factor 1 receptor
IGF-2	Insulin-like growth factor 2
IRS-1	Insulin receptor substrate 1
IRS-2	Insulin receptor substrate 2
GABA	gamma-aminobutyric acid
NC	Neuro-Cells
CD105	Cluster of differentiation 105
CD90	Cluster of differentiation 90
CD271	Cluster of differentiation 271
CD73	Cluster of differentiation 73
CD34	Cluster of differentiation 34
ALS	Amyotrophic lateral sclerosis
FDTL	Frontotemporal dementia
FUS	Fused in sarcoma protein
IL-1β	Interleukin-1β
IL-6	Interleukin-6
APP	Amyloid precursor protein
WT	Wild-type
APD	Amyloid plaque density
Egr1	Early growth response 1
Gdf15	Growth differentiation factor 15
Sirt1	Sirtuin 1
*Irs2*	Insulin receptor substrate 2 gene
*Igf*	Insulin-like growth factor gene
*Bdnf*	Brain-derived neurotrophic factor gene
*Syp*	Synaptophysin gene
*Pgc1α*	Peroxisome proliferator-activated receptor gamma coactivator 1-alpha gene
*Sqstm*	Sequestosome 1 gene
*Arc*	Activity-regulated cytoskeleton-associated protein gene
*Cldn5*	Claudin-5 gene
BBB	Blood–brain barrier
BACE-1	β-secretase 1
P62	P62 protein (alternate name for SQSTM1)
C57BL/6	C57 Black 6 strain of laboratory mouse
RNA	Ribonucleic acid
qRT-PCR	Real-time quantitative reverse transcription polymerase chain reaction
cDNA	Complementary DNA
i.c.	Intracerebral
DAPI	4′,6-diamidino-2-phenylindole
SEM	Standard error of the mean
