# Supplementary material for: Therapeutic Effects of Neuro-Cells on Amyloid Pathology, BDNF Levels, and Insulin Signalling in APPswe/PSd1E9 Mice
Source: Cells. 2025 Aug 20;14(16):1293. doi: 10.3390/cells14161293 (PMC12384073; doi:10.3390/cells14161293)
Supplement: Supplementary file 1 [file cells-14-01293-s001.zip › cells-3757382-supplementary.pdf]

Supplementary File

Supplementary Table S1. List of primers for RT-qPCR

Gene	Forward	Reverse
Gapdh	CAC TGA GCA TCT CCC TCA CA	GTG GGT GCA GCG AAC TTT AT
Gdf15	GAC TGT GCA GGC AAC TCT TG	CGA TAC AGG TGG GGA CAC TC
Sirtuin1	TTG CAA CAG CAT CTT GCC TG	CCT AGG GCA CCG AGG AAC TA
Sqstm1	ATG GTG CAC CCC AAT GTG AT	CTG CAC AGG TCG TAG TCT GG
Cldn5	GAG TTC AGC TTC CCG GTC AA	CTC CCG CCC TTA GAC ATA GTT C
Ifg1	GTA CTT CAG AAG CGA TGG GGA	AGA AGA GGT GTG AAG ACG ACA
Igf1r	AAT GCT CCA AGG ATG CAC CA	GTT CTC CAA CTC CGA GGC AA
Insr	CAG TTT GTG GAA CGG TGC TG	CAG GTC TCC ACA CAC TGA CC
Irs2	CTG CGT CCT CTC CCA AAG TG	GGG GTC ATG GGC ATG TAG C
Bdnf	CGG CGC CCA TGA AAG AAG TA	AGA CCT CTC GAA CCT GCC CT
Syp	TGC CAA CAA GAC GGA GAG TG	TAG TGC CCC CTT TAA CGC AG
Tnf	TTG TCT TAA TAA CGC TGA TTT GGT	GGG AGC AGA GGT TCA GTG AT
Pgc1	GAA TCA AGC CAC TAC AGA CAC CG	CAT CCC TCT TGA GCC TTT CGT G

Figure S1

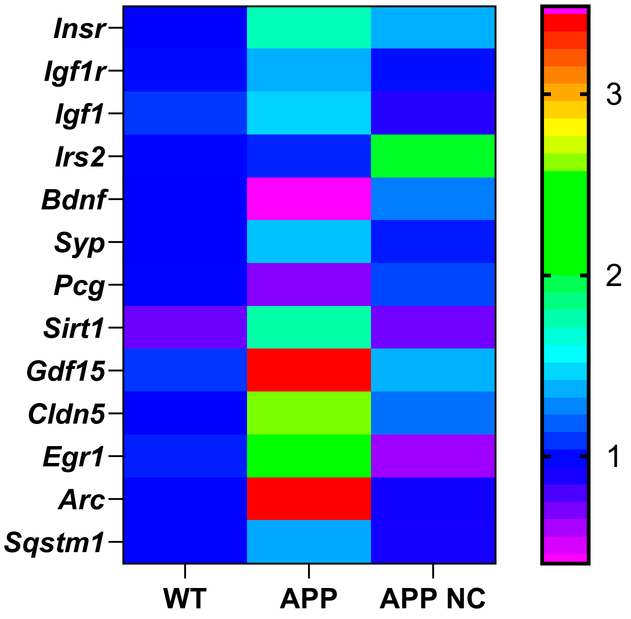

Figure S1. Heat map representation of the expression of significantly altered genes in the prefrontal cortex of WT, APP/PS1 and APP/PS1-NC mice (see ms text and Figure 5).
